# A Study of the Disruptive Effect of the Acetate Fraction of *Punica granatum* Extract on *Cryptococcus* Biofilms

**DOI:** 10.3389/fmicb.2020.568258

**Published:** 2021-01-18

**Authors:** Paulo C. M. Villis, Alessandra T. de Macedo, Haryne L. A. Furtado, Pedro H. C. Fontenelle, Ingrid S. Gonçalves, Thayariane L. Mendes, Brenda L. A. Motta, Pedro L. L. Marinho, Aruanã J. M. C. R. Pinheiro, Lídio G. Lima-Neto, Cristina A. Monteiro, Luís C. N. da Silva, Gabriella F. Ferreira, Rodrigo A. Holanda, Julliana R. A. Santos

**Affiliations:** ^1^Laboratório de Eletroquímica e Biotecnologia, Universidade Ceuma, São Luís, Brazil; ^2^Laboratório de Microbiologia Ambiental, Universidade Ceuma, São Luís, Brazil; ^3^Laboratório das Infecções do Trato Respiratório, Universidade Ceuma, São Luís, Brazil; ^4^Laboratório de Microbiologia Aplicada, Universidade Ceuma, São Luís, Brazil; ^5^Laboratório de Patogenicidade Microbiana, Universidade Ceuma, São Luís, Brazil; ^6^Programa Multicêntrico de Pós-Graduação em Bioquímica e Biologia Molecular, Departamento de Farmácia, Universidade Federal de Juiz de Fora (Campus Avançado Governador Valadares), Governador Valadares, Brazil; ^7^Laboratório de Biologia Molecular de Microrganismos Patogênicos, Universidade Ceuma, São Luís, Brazil

**Keywords:** *Cryptococcus gattii*, *Cryptococcus laurentii*, biofilm, *Punica granatum*, cryptococcosis

## Abstract

Cryptococcosis, caused by yeasts of the genus *Cryptococcus*, is an infectious disease with a worldwide distribution. *Cryptococcus neoformans* and *Cryptococcus gattii* are the species that commonly cause this disease in humans; however, infections caused by *Cryptococcus laurentii*, especially in immunocompromised patients, are increasingly being reported. Owing to the increase in the resistance of fungi to antifungals, and a lack of treatment options, it is important to seek new therapeutic alternatives such as natural products. Among these are plant species such as *Punica granatum*, which is used in folk medicine to treat various diseases. This study aimed to evaluate the activity of the acetate fraction of *P. granatum* leaf extract against environmental and clinical isolates of *Cryptococcus*. Three environmental isolates of *C. laurentii*, PMN, PMA, and PJL II, isolated from soils of different municipalities in the state of Maranhão, a clinical isolate, *C. gattii*, from a patient with neurocryptococcosis, and a standard strain of *C. gattii* (ATCC 32068) were used. The minimum and fractional inhibitory concentrations (MIC and FIC, respectively) and time-kill curve of the extract and fluconazole were determined to assess the susceptibility profile of the fungal isolates. Larvae of *Tenebrio molitor* were infected with *Cryptococcus* strains, and the effects of acetate fraction of *P. granatum* extract and fluconazole on the survival and fungal burden were determined. The extract activity was tested against pre-formed biofilms. The acetate fraction of *P. granatum* extract showed promising antifungal activity against all the species of *Cryptococcus* evaluated in this study, with an MIC value lower than that of fluconazole. The indices obtained in the FIC test indicated that the antimicrobial effect of the combination of the extract and antifungal was indifferent for 80% of the isolates. The *P. granatum* acetate fraction reduced the pre-formed biofilm of some isolates, showing better activity than fluconazole, which is consistent with results from fluorescence microscopy. This is the first study on the use of *P. granatum* and its ability to inhibit *Cryptococcus* biofilms; therefore, further studies and tests are needed to investigate the components and mechanism of action of *P. granatum* against cryptococcosis agents.

## Introduction

Cryptococcosis is a globally distributed fungal infection caused by species of the genus *Cryptococcus* (May et al., 2016), which are encapsulated yeasts found in the environment. The two most clinically prevalent species in humans are *Cryptococcus neoformans* and *Cryptococcus gattii* (Maziarz and Perfect, 2016; Castro-Láinez et al., 2019); however, there has been an increase in cryptococcal infections caused by an unusual species, *Cryptococcus laurentii* (Santos et al., 2016).

Such infections occur through the inhalation of propagules present in the environment. These yeasts can cause lung infection and show tropism in the central nervous system (CNS), which can cause meningoencephalitis, the severe form of the disease. Cryptococcal species have several virulence factors which allow them to progress from latent infection to disease in immunocompetent individuals as well as in immunosuppressed patients, especially those with HIV/AIDS (Chen et al., 2017).

*Cryptococcus laurentii* is a rare human pathogen which was previously considered saprophytic and non-pathogenic. Infections caused by this fungus, especially in immunocompromised patients, have been reported (Gupta et al., 2018). *Cryptococcus laurentii* and *Cryptococcus albidus* are responsible for 80% of cases other than those of *C. neoformans* and *C. gattii*. They are capable of causing localized and systemic infections in humans, and their clinical manifestations vary from skin lesions to fungemia (Ajesh and Sreejith, 2012).

*Cryptococcus* spp. present several virulence factors that contribute to the spread and permanence of the fungus in the body. The main ones include the ability to grow at the temperature of the human body; a polysaccharide capsule which prevents phagocytosis; the production of enzymes such as laccase, superoxide dismutase, phospholipase, and urease; and biofilm formation (Tavares et al., 2019).

Biofilms, which are rich in extracellular polymeric matrices (EPMs), provide protection against phagocyte activity in tissues, and make cryptococcal cells resistant to standard antifungal therapy (Jabra-Rizk et al., 2004). The biofilm of *Cryptococcus* spp. is highly resistant to azole antifungals, whereas amphotericin B (AMB) and its lipid formulations show good efficacy; however, the effective concentrations are above the therapeutic range, and hence cause severe toxicity and renal dysfunction (Kumari et al., 2017).

For the clinical treatment of cryptococcosis, the combination of AMB and 5-fluorocytosine (5FC) is prescribed, although the latter is not available in underdeveloped countries such as Brazil, followed by a maintenance period with fluconazole (Worasilchai et al., 2017). The indiscriminate use of azoles and lack of patient adherence to the correct treatment have promoted an increase in resistant clinical isolates (Rêgo et al., 2019). Therefore, it is necessary to search for new molecules with antifungal activity. To this end, extracts of natural products are suitable alternatives owing to their low toxicity and high antimicrobial activity. Several studies have shown that plants are rich in active molecules that exhibit medicinal properties (Akroum, 2017).

*Punica granatum* is a plant that originated in the Middle East but is currently grown across the Mediterranean, China, India, South Africa, and America. It is used in folk medicine to treat various diseases such as ulcers, fever, diarrhea, and microbial infections (Endo et al., 2010). In addition, different parts of *P. granatum*, such as the leaves, flowers, and seeds, have antimicrobial, antioxidant, and antifungal properties (Lavaee et al., 2018). The antifungal activity of *Punica granatum* extract has already been reported against some species of *Candida* (Anibal et al., 2013; Mansourian et al., 2014; Akroum, 2017; Paul et al., 2018) and against dermatophyte fungi (Foss et al., 2014).

The occurrence of infectious diseases is directly linked to the environmental conditions to which people are exposed. Hence, it is essential to design studies that consider the relationship between urban planning, the development of diseases and systemic infections, and above all, the environmental relevance of using plant extracts for the treatment of fungal diseases. Therefore, this study aimed to evaluate the activity of the acetate fraction of *P. granatum* leaf extract against clinical and reference isolates of *C. gattii* and environmental isolates of *C. laurentii*.

## Materials and Methods

### Ethical Aspects

The studies involving human participants were reviewed and approved by the research ethics committee of the CEUMA University (approval number: 2.927.864), considering all ethical aspects that involve the use of human participants, according to Resolution 466/12 of the National Health Council. The participant provided their written informed consent to participate in this study.

### Strains

The standard strain of *C. gattii* (ATCC 32068), three environmental isolates of *C. laurentii* from soils of different municipalities in the State of Maranhão (PMN, PMA, and PJL II), and a clinical isolate of *C. gattii* from a patient with neurocryptococcosis (CEP n° 2.927.864) were obtained, and stored in the Culture collection of the Laboratory of Environmental Microbiology of UNICEUMA.

These samples were seeded and grown on Sabouraud Dextrose Agar (SDA), at a temperature of 28°C for the environmental samples and 37°C for the clinical samples, for a period of 48 h.

### Preparation of Acetate Fraction and Antifungals

The leaves of *P. granatum* were obtained from the Herbarium of the Federal University of Maranhão (UFMA) in São Luís - Maranhão, Brazil. They were completely dried at 40°C in a greenhouse, and crushed to obtain a powder, which was mixed with 70% alcohol and occasionally shaken for 7 days at room temperature. The ethyl acetate fraction of pomegranate leaves were obtained as previously described (Marques et al., 2016). The acetate fraction of the lyophilized extract, which was obtained *via* evaporation and drying, was kindly provided by Prof. Dr. Lídio Gonçalves Lima Neto (UNICEUMA).

For the antifungal microdilution test, the acetate powder fraction was diluted in distilled water to a concentration of 1000 μg/mL.

### Minimum Inhibitory Concentration (MIC)

The minimum inhibitory concentration (MIC) was determined using a microdilution test in 96-well microplates (Clinical and Laboratory Standards Institute, 2008). The inoculants were prepared from the isolates grown on SDA, by suspending them in 4 mL of sterile saline and comparing them with a McFarland scale of 0.5 × 10^6^ CFU/mL. Then, they were diluted in RPMI medium to a concentration of 0.5 × 10^3^ CFU/mL. Initially, 100 μL of RPMI medium was placed in each well; then, 100 μL of extract was added in the 2nd column alone, whereas serial dilution was performed up to the 11th column, to prepare a concentration range of 500 to 0.97 μg/mL. Thereafter, 100 μL inoculum was added to each well, with the exception of the 12th column, which represented the sterility control. The microplates were incubated at 28 or 37°C for 72 h. After the incubation period, a visual reading was performed to determine the MIC. The viability dye, resazurin, was also added to confirm the results.

### Time-Kill Curves

An assay was performed to evaluate the time-kill kinetics of the acetate fraction of *P. granatum* extract and fluconazole alone or in combination against *Cryptococcus*. For fluconazole (FLU) and the extract (EXT), the tested concentrations were equal to the MIC, twice the MIC (2× the MIC), and four times the MIC (4× the MIC) for each strain. In combination, EXT and FLU at MIC, twice the MIC (2× the MIC), and four times the MIC (4× the MIC) were tested. A 100-μl inoculum of *Cryptococcus* strain was placed on microtiter plates containing antifungal agent alone or in combination. In sequence, the results were determined by plating an amount of 10 μL from each well on SDA, followed by incubation at 37°C (clinical isolates) and 28°C (environmental isolates) at different intervals for 72 h prior to colony counting. After the incubation period, the CFU count was performed (Santos et al., 2012).

### Fractional Inhibitory Concentration (FIC)

The fractional inhibitory concentration (FIC) values were determined using a checkerboard microdilution, in which fluconazole (FLU) and the extract (EXT) were tested in combination. Antifungal concentrations varied from 0.25 to 128 μg/mL.

RPMI medium and RPMI medium + inoculum were added to the sterility and growth control wells, respectively. Fifty microliters of RPMI medium was placed in each well; then, the extract was added and dilution of the 2nd to 11th columns was started. In the 2nd column, an additional 37.5 μL of RPMI medium, followed by 12.5 μL of extract, made up to 100 μL, was added. Finally, 10 μL of fungal inoculum was added to each well, with the exception of the sterility control wells. The samples were incubated at 28°C or 37°C, in the range of 16 to 20 h.

Then, the FIC was determined using the formula:

$$F\,I\,C=\frac{M\,I\,C\,F\,l\,u\,c\,o\,n\,a\,z\,o\,l\,e\,i\,n\,c\,o\,m\,b\,i\,n\,a\,t\,i\,o\,n}{M\,I\,C\,F\,l\,u\,c\,o\,n\,a\,z\,o\,l\,e}+\frac{M\,I\,C\,E\,x\,t\,r\,a\,c\,t\,i\,n\,c\,o\,m\,b\,i\,n\,a\,t\,i\,o\,n}{M\,I\,C\,E\,x\,t\,r\,a\,c\,t}$$

The index was calculated for all combinations, and the results were expressed as the arithmetic means of the FIC. The interaction was classified as synergism for FIC ≤ 0.5, indifferent for 0.5 > FIC ≤ 4.0, and antagonism for FIC > 4.0 (Odds, 2003).

### Activity of Acetate Fraction of *P. granatum* Extract Against Pre-formed Biofilm

Biofilms were prepared according to the methodology of Brilhante et al. (2015) and Kumari et al. (2017), with modifications. Firstly, the samples were cultured on plates containing Sabouraud agar and incubated at 37°C for 48 h. After subculturing in Sabouraud broth, and incubation at 37°C for 24 h, the sample suspensions were centrifuged at 3000 rpm for 10 min; then, the supernatants were discarded, and the pellets were washed twice with PBS. Subsequently, the cells were re-suspended in RPMI medium to obtain a concentration of 1 × 10^6^ cells/mL. Tests were performed in triplicate in 96-well plates, wherein, initially, 100 μL of the fungal suspension was added to 100 μL of RPMI medium and incubated at 37°C for 48 h; soon after, the samples were exposed to 100 μL of the extract and 100 μL of fluconazole and incubated at 37°C for 48 h. Then, the plates were washed twice with PBS to remove non-adhered cells. To evaluate the viability of the biofilm, 100 μL of MTT was added, followed by incubation at 37°C for 4 h, and a reading was performed using a spectrophotometer at 492 nm.

### Fluorescence Microscopy Assay

The fluorescence microscopy assay was performed in duplicate, according to the methodology of Jurcisek et al. (2011) with modifications. A 24-well plates containing coverslips were used. The samples *C. laurentii* PMA, *C. gattii* 32068 ATCC, and *C. gattii* CASA were previously grown on SDA for 72 h. Inocula were adjusted to 1 to 5 × 10^6^ cells/mL. A 500 μL of inoculum was added to each well, and 500 μL of RPMI in the well corresponding to the negative control. The plates were incubated for an hour and a half at 37°C (clinical strain and ATCC) and 28°C (environmental strain). Subsequently, the wells were washed twice with PBS and 500 μL of RPMI was added. The plates were incubated for 24 h. After the incubation period, 500 μL of the acetate fraction of *P. granatum* extract, fluconazole and combination at MIC, 2× MIC, 4× MIC concentrations were added. Then, he plates were incubated again for 24 h. They were washed twice with PBS, fixed with 500 μL of methanol for 10 min, and finally, the coverslips were stained with Acridine Orange (10 μg/mL) and mounted on slides. The reading was performed under a fluorescence microscope (Imager Z2, ZEISS, Germain). The sum of fluorescences was used to evaluate disturbances on biofilm. Assays were performed in triplicate.

### Survival Curve and Quantification of Fungal Burden in Larvae of *Tenebrio molitor*

Larvae of *Tenebrio molitor* were used in all experiments. Larvae were inoculated with 10 μL of 10^3^ (*C. gattii* CASA) or 10^5^CFU/larvae (*C. laurentii* PMA, PJLII, PMN and ATCC) or PBS only (control). To verify efficacy of treatment, fluconazole and/or acetate fraction of *P. granatum* extract (MIC) was administered once from 1 day post infection (d.p.i.) Larvae were monitored twice daily for survival (de Souza et al., 2015).

Groups of larvae of *Tenebrio molitor* (*n* = 10/group) were inoculated with 10^3^ (*C. gattii* CASA) or 10^5^CFU/larvae (*C. laurentii* PMA, PJLII, PMN, and ATCC 32068) or PBS only (control) to obtain larvae homogenates in 1 mL of PBS at 3 days post inoculation. The larvae homogenates were plated onto SDA with chloramphenicol for the determination of colony forming units (CFU) as described (de Souza et al., 2015).

### Statistical Analysis of Data

The results are presented as mean ± standard deviation. Statistical analyses were performed using version 5.0 of GraphPad Prism (GraphPad Software, San Diego, CA, United States). The Student’s non-parametric *t*-test and analysis of variance (ANOVA) were used. Survival curve was plotted by Kaplan Meier analysis and results were analyzed using the log rank test. A significance level of 95% was considered to differentiate the measurements (*p* < 0.05).

## Results

### Minimum Inhibitory Concentration and Time-Kill Curves

The acetate fraction of *P. granatum* showed antifungal activity against all the species of *Cryptococcus* tested in this study (Table 1).

**TABLE 1:** Minimum inhibitory concentration (MIC) of acetate fraction of *P. granatum* and fluconazole against isolates of *Cryptococcus* spp.

	MIC (μg/mL)	
	
Strain	*P. granatum*	Fluconazole
PNM (*C. laurentii*)	1.95	32
PMA (*C. laurentii*)	1.95	32
PJL II (*C. laurentii*)	1.95	32
*Cryptococcus gattii* I.C.	15.62	32
ATCC 32068 (*C. gattii*)	31.25	32

**P., Punica;* C.I., clinical isolate.*

For the *C. gattii* clinical isolate, the acetate fraction of *P. granatum* presented a MIC value of 15.62 μg/mL, whereas for samples of *C. laurentii* (PMA, PJL II, and PNM) and *C. gattii* (ATCC 32068), it exhibited MIC values of 1.95 and 31.25 μg/mL, respectively. All the isolates had a MIC with respect to fluconazole (32 μg/mL).

**FIGURE 1:**
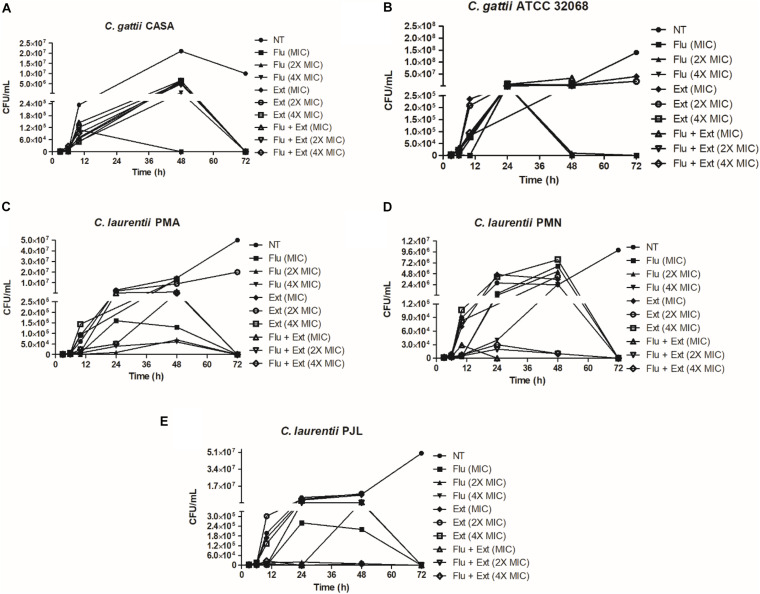
Time-kill curves of fluconazole and acetate fraction of *P. granatum* extract alone or in combination against *Cryptococcus* strains. Time-kill curve were performed with fluconazole or acetate fraction of *P. granatum* extract at 1× MIC, 2× the MIC, and 4× the MIC. **(A)** Time-kill curve performed against *C. gattii* CASA. **(B)** Time-kill curve performed against *C. gattii* ATCC 36068. **(C)** Time-kill curve performed against *C. laurentii* PMA. **(D)** Time-kill curve performed against *C. laurentii* PMN. **(E)** Time-kill curve performed against *C. laurentii* PJL.

To evaluate the kinetic of the action of the acetate fraction of *P. granatum* and the antifungal tested, the assays of time-kill curves were performed (Figure 1). For FLU and/or extract against *C. gattii* CASA (clinical strain, Figure 1A), reduction of CFU occurred only after 48 h. FLU and/or extract against *C. gattii* ATCC 32068, increased this period to 72 h (Figure 1B). When the tests were carried out with fluconazole and/or extract against *C. laurentii* strains, *C. laurentii* PMA (Figure 1C), *C. laurentii* PMN (Figure 1D), *C. laurentii* PJL (Figure 1E), the profile of reduction of the CFU was slower for each strain. It was possible to observe a reduction after 72 h.

### Fractional Inhibitory Concentration

A comparison of the test results showed that one sample exhibited growth at all concentrations tested, i.e., it was resistant to all the concentrations evaluated. The indices obtained indicated that the antimicrobial effect of the combination of the acetate fraction of *P. granatum* and antifungal (fluconazole) was indifferent for most of the samples tested, with 1 > FIC < 4, whereas the interaction was antagonistic against only one of the isolates of *C. laurentii* (PNM) (Table 2).

**TABLE 2:** Fractional inhibitory concentration (FIC) of acetate fraction of *P. granatum* extract against *Cryptococcus* isolates.

Isolated	FIC average	Type of interaction
PMN (*C. laurentii*)	8.2	Antagonism
PMA (*C. laurentii*)	–	Indifferent
PJL II (*C. laurentii*)	3.23	Indifferent
*Cryptococcus gattii* I.C.	3.73	Indifferent
ATCC 32068 (*C. gattii*)	1.23	Indifferent

*C.I., clinical isolate.*

### Activity of Acetate Fraction of *P. granatum* Extract Against Pre-formed Biofilm

**FIGURE 2:**
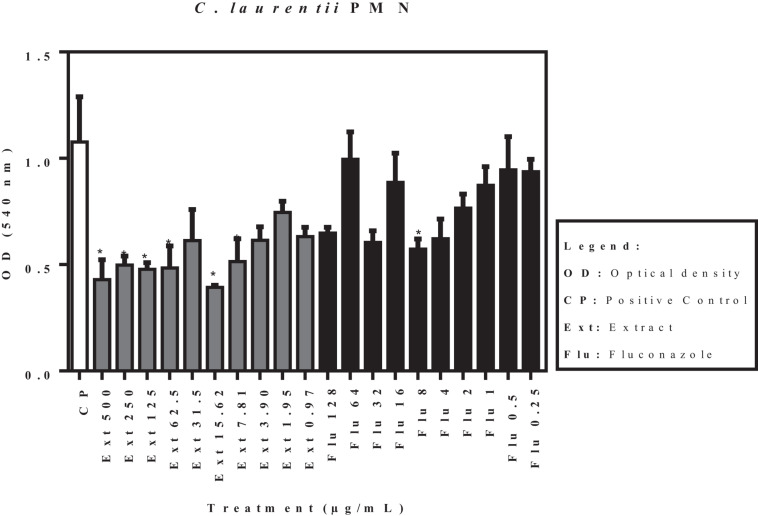
Activity of acetate fraction of *P. granatum* extract and fluconazole against pre-formed biofilm of *C. laurentii* PMN. *p < 0.05. Assays were performed in duplicate.

Figure 2 shows the activity of the acetate fraction of *P. granatum* extract and fluconazole against a pre-formed biofilm of *C. laurentii* PMN. Six of the ten concentrations of the acetate fraction showed significant activity, whereas only one concentration of fluconazole (8 μg/mL) showed significant activity when compared to the positive control.

**FIGURE 3:**
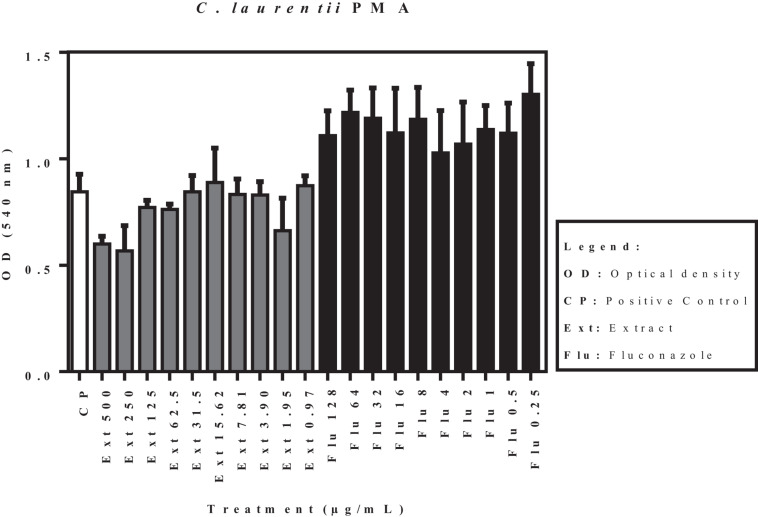
Activity of acetate fraction of *P. granatum* extract and fluconazole against pre-formed biofilm of *C. laurentii* PMA. Assays were performed in duplicate.

**FIGURE 4:**
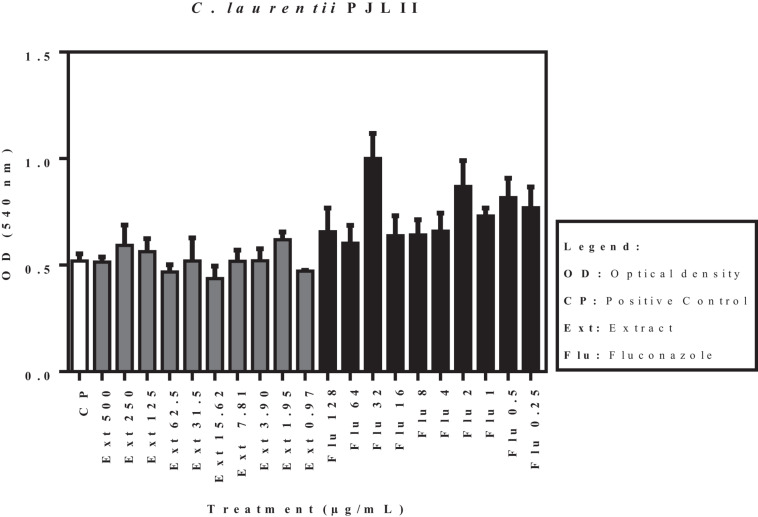
Activity of acetate fraction of *P. granatum* extract and fluconazole against pre-formed biofilm of *C. laurentii* PJL II. Assays were performed in duplicate.

**FIGURE 5:**
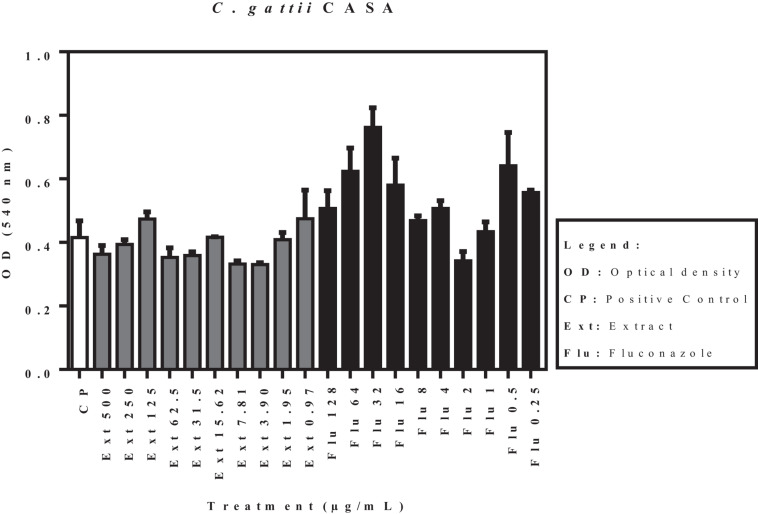
Activity of acetate fraction of *P. granatum* extract and fluconazole against pre-formed biofilm of *C. gattii.* Assays were performed in duplicate.

The effects of the acetate fraction of *P. granatum* extract and fluconazole on the isolates (*C. laurentii* PMA and PJL II, and *C. gattii*), reflected by the optical density, are shown in Figures 3–5. According to the statistical analysis carried out on the pre-formed biofilms, none of the samples showed a significant difference (*p* < 0.05) when compared to the positive control.

**FIGURE 6:**
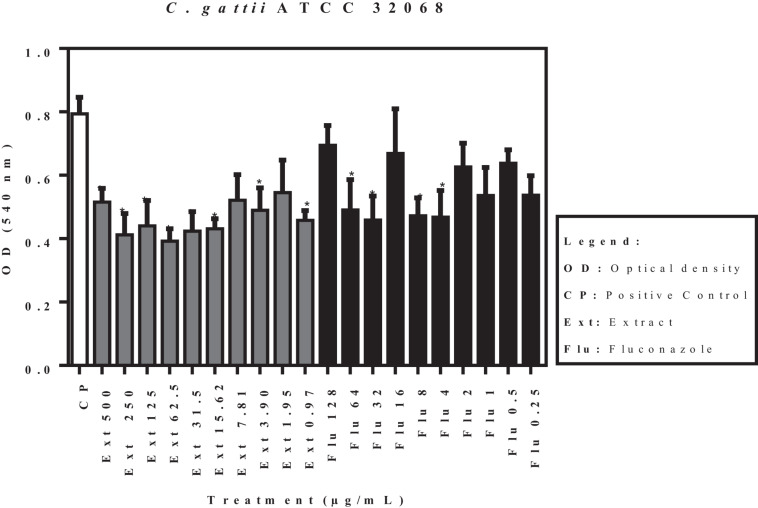
Activity of acetate fraction of *P. granatum* extract and fluconazole against pre-formed biofilm of *C. gattii* (ATCC 32068). *p < 0.05. Assays were performed in duplicate.

Figure 6 shows the activity of the acetate fraction of *P. granatum* extract and fluconazole against a pre-formed biofilm of *C. gattii* (ATCC 32068). Of the ten concentrations of the acetate fraction that were tested, eight presented significant activity. Concentrations of 7.81 and 1.91 μg/mL were not statistically significant when compared to the control. On the contrary, only four concentrations of fluconazole showed significant activity compared to the positive control.

### Fluorescence Microscope

**FIGURE 7:**
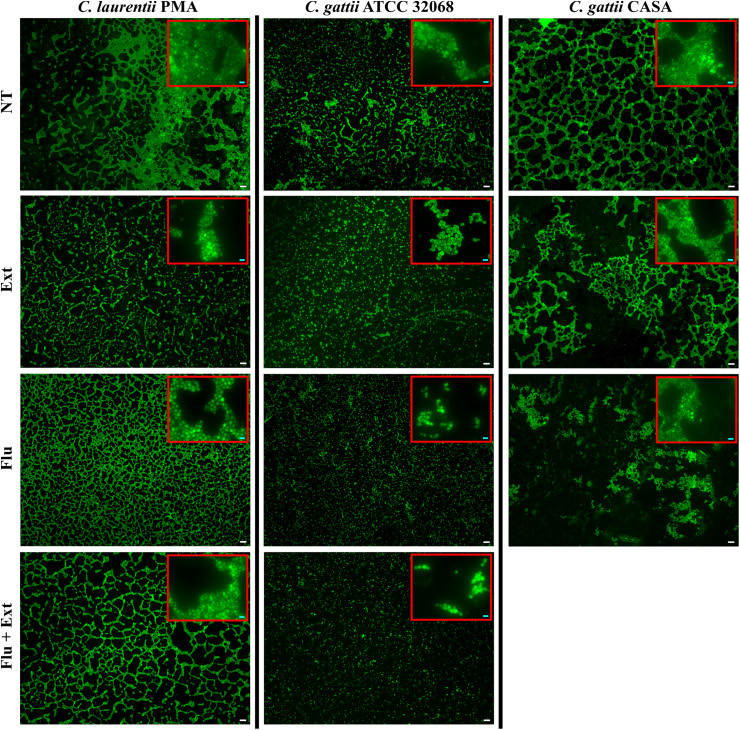
Aspects of biofilms of *Cryptococcus* spp. after treatment with acetate fraction of *P. granatum* extract and/or fluconazole. Yeast cells were stained with 10 μg/mL acridine orange and visualized at 50× and 400× magnification (within red rectangles). Disturbances on biofilms occurred at equal or greater MIC when compared with non-treated cells, as follow: *C*. *laurentii* PMA at 4× MIC for Ext or Flu, and 4× MIC for Flu + Ext; *C*. *gattii* ATCC 32068 at 1× MIC for Ext or Flu, and 1× MIC for Flu + Ext; and *C. gattii* CASA at 2× MIC for Ext or Flu. The sum of fluorescences was used to evaluate disturbances on biofilm. Assays were performed in triplicate. “NT,” “Ext,” “Flu,” and “Flu + Ext” mean non-treated; acetate fraction of *P. granatum* extract, fluconazole and combination between them, respectively. MIC mean minimal inhibitory concentration. White (50 μm) and bright blue (5 μm) bars.

Yeast cells were stained with 10 μg/mL acridine orange and visualized at 50× and 400× magnification (within red rectangles). Disturbances on biofilms occurred at equal or greater MIC and/or higher concentrations when compared with non-treated cells, as follow: *C*. *laurentii* PMA at 4× MIC for Ext or Flu, and 4× MIC for Flu + Ext; *C*. *gattii* ATCC 38068 at 1× MIC for Ext or Flu, and 1× MIC for Flu + Ext; and *C. gattii* CASA at 2× MIC for Ext or Flu (Figure 7).

### Survival and CFU/g of Larvae

**FIGURE 8:**
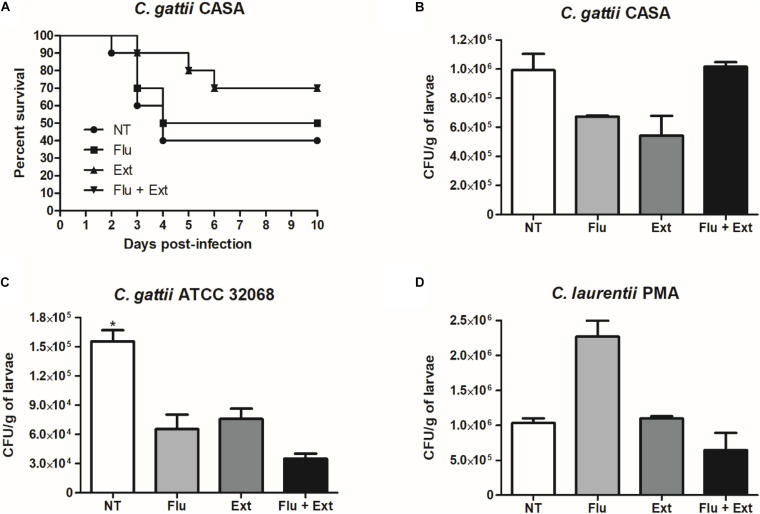
Larvae (*n* = 10) were monitored daily for the survival curve **(A)**. Fungal burden in larvae infected with the *C. gattii* CASA strain and treated with fluconazole, acetate fraction of *P. granatum* extract or combination **(B)**. Fungal burden in larvae infected with the *C. gattii* ATCC 32068 strain and treated with fluconazole, acetate fraction of *P. granatum* extract or combination **(C)**. Fungal burden in larvae infected with the *C. laurentii* PMA strain and treated with fluconazole, acetate fraction of *P. granatum* extract or combination **(D)**. NT, non-treated group; Flu, fluconazole; Ext, extract. *p < 0.05.

The median survival in the group infected and non-treated (NT) with the *C. gattii* CASA (clinical strain) was 4 days versus 7 days for the group infected with this strain and treated with fluconazole (Figure 8A). Acetate fraction of *P. granatum* extract alone or in combination with fluconazole increased survival of larvae infected with *C. gattii* CASA compared to the non-treated larvae (NT) or treated with fluconazole alone (Figure 8A). On the other hand, larvae infected with the *C. gattii* ATCC or *C. laurentii* strains and treated or not treated survived for 10 days post-inoculation (data not shown).

In addition, acetate fraction of *P. granatum* extract or fluconazole (MIC) reduced the fungal burden in the larvae infected with *C. gattii* CASA compared to non-treated larvae (Figure 8B). Acetate fraction of *P. granatum* extract and/or fluconazole (MIC) reduced the fungal burden in the larvae infected with *C. gattii* ATCC 32068 (*p* < 0.05) compared to non-treated larvae (Figure 8C). However, no reduction in CFU/g of larvae was observed when infected with *C. laurentii* PMA and treated with fluconazole or extract alone, but the combination showed a tendency to reduce the fungal burden in comparison to the non-treated group (Figure 8D).

## Discussion

The indiscriminate use of antimicrobial drugs has been growing steadily owing to a lack of knowledge about the damage that such use can cause. Therefore, the use of medicinal plants has been gaining ground due to their antimicrobial, anti-inflammatory, and antioxidant activity against fungi and bacteria (Dos Santos et al., 2017).

According to Brandão et al. (2014), several studies have been carried out on the use of plants with medicinal properties, as therapeutic alternatives, in view of the development of resistance by groups of microorganisms; however, there is no standard regarding the formulation of the concentrations or the type of technique to be used.

Owing to research in the area of phytopharmaceuticals, approximately 350 000 species of plants with antimicrobial characteristics have been identified in Brazilian Universities, showing that there is potential for natural products in medicinal therapy (Pinto et al., 2013).

According to Silva et al. (2013), the plant species *P. granatum*, popularly known as Romã, Romãzeira, or Romeira, is used by many, owing to its therapeutic properties as well as antimicrobial and anti-inflammatory activity. Frias and Kozusnyandreani (2010) verified these characteristics through phytochemical analysis of extracts linked to secondary metabolites such as tannins and flavonoids, which promote fungicidal or fungistatic capacity. In a study using animal models, Pinheiro et al. (2018, 2019) found that the galoyl-HHDP-glucose molecule, which is present in *P. granatum* leaves, may have be one of the compounds associated with anti-inflammatory effects, which were previously investigated using treatments with hydro-alcoholic extracts and ethyl acetate fractions of *P. granatum*, wherein the fraction was effective in reducing lung inflammation.

According to Marques et al. (2016), and Pinheiro et al. (2018, 2019), the extract shows no toxicity either in an LPS-induced acute lung injury mouse model or in cellular cytotoxicity assays at the concentrations used in this study. These characteristics confirm that the studied compound can impart benefits to certain therapeutic approaches, considering that the use of antimicrobials has been increasing dramatically, and is causing the development of resistance in clinical isolates, i.e., environmental fungi of clinical interest. Therefore, studies on the activity of this extract are important, since there are no reports on the effect of *P. granatum* extracts on *Cryptococcus* spp., to evaluate the antifungal potential of *P. granatum* leaf extracts against these yeasts.

In our study, the indices obtained indicated that the antimicrobial effect of the combination of the acetate fraction and antifungal was indifferent for most of the tested isolates. It is important to note that the choice of fluconazole was based on its use in the treatment of diseases caused by yeasts, mainly in immunocompromised patients (Mukherjee et al., 2005).

The susceptibility to acetate fraction of *P. granatum* extract and fluconazole alone or in combination were confirmed with lower fluorescence intensity for biofilms of *C. gattii* CASA (clinical strain) and *C. gattii* ATCC in comparison to non-treated cells, and in the *in vivo* tests in larvae of *Tenebrio molitor*. Also, the susceptibility to acetate fraction of *P. granatum* alone or in combination with fluconazole for *C. laurentii* PMA was confirmed by lower fluorescence intensity compared to non-treated cells. On the other hand, the reduced susceptibility to fluconazole was confirmed with higher fluorescence intensity for *C. laurentii* PMA in comparison to non-treated cells, and in the *in vivo* tests in larvae of *Tenebrio molitor*, as this drug was unable to reduce the CFU in the larvae. Additionally, environmental isolates of fluconazole-resistant *Cryptococcus* have also been reported (Chowdhary et al., 2011).

In light of the scarcity of new classes of antimicrobial drugs or different targets of action, combinations of phyto-therapeutic and antimicrobial products are considered as accessible strategies for the treatment of patients affected by diseases caused by fungi; such combinations will increase the number of molecular targets against which current agents are effective (Kumari et al., 2017).

Although fluconazole in combination with the acetate fraction of *P. granatum* extracts had different effects on samples of *Cryptococcus* spp., the MIC values and suggested a potential for inhibiting antifungal growth. Also, the time-kill curves confirmed the kinetics of growth and the dynamism of the action of the acetate fraction of *P. granatum* extract and fluconazole either alone or in combination. In addition, our survival and CFU data confirmed the potential efficacy of acetate fraction of *P. granatum* extract in the outcome of antifungal therapy of cryptococcosis.

Due to the increasing number of cases of diseases caused by fungi in nosocomial environments, and the emergence of new strains resistant to antimicrobials, mainly associated with the ability to form biofilms, it has become essential to develop new classes of drugs with low phyto-therapeutic resistance (Pessoa et al., 2012). The acetate fraction of *P. granatum* used in this study did not demonstrate anti-biofilm activity at some concentrations when compared to the results of Cavaleiro et al. (2015) and Cardoso et al. (2016) which indicated a strong antifungal activity of the extracts of *Ocimum basilicum* and *Angelica major* against samples of *C. neoformans* and potential anti-biofilm activity against *Candida* spp.

It is important to highlight that there are still controversies regarding the biofilm formation mechanism of *Cryptococcus* spp. The ability to form biofilms is evident in environmental isolates, and may be related to other virulence factors such as the yeast polysaccharide capsule, especially when they are subjected to environmental conditions, confirming that *in vitro* cryptococcal biofilms are less susceptible to antifungal agents in these circumstances (Ravi et al., 2009). In addition, some authors have mentioned that the development of biofilms from isolates of *C. neoformans* depends on other characteristics such as capsular production, the physical properties of the substrate, and the environmental conditions; therefore, such aspects of microbial biofilms may be important for the administration of therapies against diseases related to fungal biofilms (Martinez and Casadevall, 2007).

**FIGURE 9:**
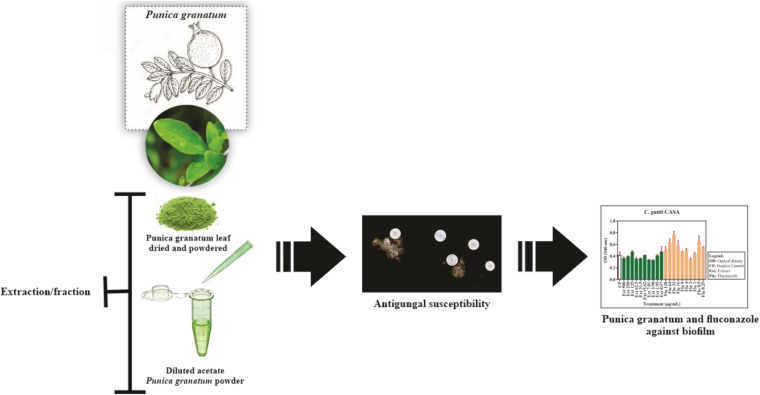
New insights into the potential antibiofilm activity of acetate fraction of *Punica granatum* against clinical and environmental isolates of *Cryptococcus*.

This is the first study on the anti-biofilm capacity of the acetate fraction of *P. granatum* against *Cryptococcus* isolates (Figure 9). However, further studies are needed to determine the active components of *P. granatum* and explain the mechanism of action against fungi that affect immunocompetent and immunocompromised individuals. These studies will complement the reduction in the use of antimicrobial drugs in the fight against fungal diseases, and highlight the importance of using herbal medicines in their treatment, particularly in a country like Brazil, where the flora are abundant and can be harnessed to address the current scenario of antimicrobial use.

## Data Availability Statement

The raw data supporting the conclusions of this article will be made available by the authors, without undue reservation.

## Ethics Statement

The studies involving human participants were reviewed and approved by the research ethics committee of the CEUMA University (approval number: 2.927.864), considering all ethical aspects that involve the use of human participants, according to Resolution 466/12 of the National Health Council. The participant provided their written informed consent to participate in this study.

## Author Contributions

PV and JS designed the study. AM, IG, PF, HF, TM, BM, PM, and AP developed the method. LN, CM, LS, GF, RH, and JS conducted the analysis and critically revised the manuscript. PV, AM, PF, HF, TM, and JS wrote the manuscript. All authors approved the final version of the manuscript.

## Conflict of Interest

The authors declare that the research was conducted in the absence of any commercial or financial relationships that could be construed as a potential conflict of interest.

## References

[B1] Ajesh K. Sreejith K. (2012). *Cryptococcus laurentii* biofilms: structure, development and antifungal drug resistance. *Mycopathologia* 174:409. 10.1007/s11046-012-9575-2 22936102

[B2] Akroum S. (2017). Antifungal activity of acetone extracts from *Punica granatum* L., *Quercus suber* L. and *Vicia faba* L. *J. Mycol. Méd.* 27 83–89. 10.1016/j.mycmed.2016.10.004 27856170

[B3] Anibal P. C. Peixoto I. T. Foglio M. A. Höfling J. F. (2013). Antifungal activity of the ethanolic extracts of *Punica granatum* L. and evaluation of the morphological and structural modifications of its compounds upon the cells of *Candida* spp. *Braz. J. Microbiol.* 44 839–848. 10.1590/S1517-83822013005000060 24516425 PMC3910198

[B4] Brandão D. O. Fernandes F. H. A. Ramos Júnior F. J. L. Silva P. C. D. Santana C. P. De Medeiros F. D. (2014). Validation of UPLC method for determination of gallic acid from *Ximenia americana* L. *Planta Med.* 80:P2O60 10.1055/s-0034-1395050

[B5] Brilhante R. S. N. Caetano E. P. Oliveira J. S. Castelo-Branco D. S. C. M. Souza E. R. Y. Alencar L. P. (2015). Simvastatin inhibits planktonic cells and biofilms of *Candida* and *Cryptococcus* species. *Braz. J. Infect. Dis.* 19 459–465. 10.1016/j.bjid.2015.06.001 26119850 PMC9427464

[B6] Cardoso N. R. Alviano C. S. Blank A. F. Romanos M. T. V. Fonseca B. Rozental S. (2016). Synergism effect of the essential oil from *Ocimum basilicum* var. Maria Bonita and its major components with fluconazole and its influence on ergosterol biosynthesis. *Evid. Based Complement. Altern. Med.* 69 241–248. 10.1155/2016/5647182 27274752 PMC4871963

[B7] Castro-Láinez M. T. Deliz-Aguirre R. Antunez D. Marco Cruz-Codina M. Cahuayme-Zuniga L. Vitale K. (2019). *Cryptococcus laurentii* meningitis in a non-HIV patient ⋆. *IDCases* 18:e00612. 10.1016/j.idcr.2019.e00612 31463196 PMC6710230

[B8] Cavaleiro C. Salgueiro L. Gonçalves M. J. Hrimpeng K. Pinto J. Pinto E. (2015). Antifungal activity of the essential oil of *Angelica major* against *Candida*, *Cryptococcus*, *Aspergillus* and dermatophyte species. *J. Nat. Med.* 69 241–248. 10.1007/s11418-014-0884-2 25576097

[B9] Chen Y. Farrer R. A. Giamberardino C. Sakthikumar S. Jones A. Yang T. (2017). Microevolution of serial clinical isolates of *Cryptococcus neoformans* var. *grubii* and *C. gattii*. *mBio* 8:e00166-17. 10.1128/mBio.00166-17 PMC5340869 28270580

[B10] Chowdhary A. Randhawa H. S. Sundar G. Kathuria S. Prakash A. (2011). In vitro antifungal susceptibility profiles and genotypes of 308 clinical and environmental isolates of *Cryptococcus neoformans* var.grubii and *Cryptococcus gattii* serotype B from North-Western India. *J. Med. Microbiol.* 60 961–967. 10.1099/jmm.0.029025-0 21393452

[B11] Clinical and Laboratory Standards Institute (2008). *Reference Method for Broth Dilution Antifungal Susceptibility Testing of Yeasts. Approved Standard M27-A3*, 3rd Edn, Wayne, PA: Clinical and Laboratory Standards Institute.

[B12] de Souza P. C. Morey A. T. Castanheira G. M. Bocate K. P. Panagio L. A. Nazareth L. (2015). *Tenebrio molitor* (Coleoptera: tenebrionidae) as na alternative host to study fungal infections. *J. Microbiol. Methods* 17 365–375. 10.1016/j.mimet.2015.10.004 26453946

[B13] Dos Santos J. E. F. Junior A. A. S. Barbosa R. N. Santos A. C. S. Lopes D. H. G. De Oliveira N. T. (2017). In Vitro antifungal activity of medicinal plants against yeasts isolated from vaginal secretion. *Sabios Rev. Saúde Biol.* 11 34–44.

[B14] Endo E. H. Cortez D. A. G. Ueda-Nakamura T. Nakamura C. V. Filho B. P. D. (2010). Potent antifungal activity of extracts and pure compound isolated from pomegranate peels and synergism with fluconazole against *Candida albicans*. *Res. Microbiol.* 161 534–540. 20541606 10.1016/j.resmic.2010.05.002

[B15] Foss S. R. Nakamura C. V. Ueda-Nakamura T. Cortez D. A. Endo E. H. Dias Filho B. P. (2014). Antifungal activity of pomegranate peel extract and isolated compound punicalagin against dermatophytes. *Ann. Clin. Microbiol. Antimicrob.* 13:32. 10.1186/s12941-014-0032-6 25260038 PMC4353666

[B16] Frias D. F. R. Kozusnyandreani D. I. (2010). Use of extracts of medicinal plants and *Eucaliptus* oil in the in vitro control of *Microsporum canis*. *Rev. Cubana Plant. Med.* 15 119–125.

[B17] Gupta M. Mishra A. K. Singh S. K. (2018). *Cryptococcus laurentii* fungemia in a low birth weight preterm neonate: India. *J. Infect. Public Health* 11 896–897. 10.1016/j.jiph.2018.04.012 29731340

[B18] Jabra-Rizk M. A. Falkler W. A. Meiller T. F. (2004). Biofilmes de fungos e resistência a medicamentos. *Doenças Infecc. Emerg.* 10 14–19. 10.3201/eid1001.030119 15078591 PMC3031105

[B19] Jurcisek J. A. Dickson A. C. Bruggeman M. E. Bakaletz L. O. (2011). In vitro biofilm formation in an 8-well chamber slide. *J. Vis. Exp.* 47:2481. 10.3791/2481 21304464 PMC3182645

[B20] Kumari P. Mishra R. Arora N. Chatrath A. Gangwar R. Roy P. (2017). Antifungal and anti-biofilm activity of essential oil active components against *Cryptococcus neoformans* and *Cryptococcus laurentii*. *Front. Microbiol.* 8:2161. 10.3389/fmicb.2017.02161 29163441 PMC5681911

[B21] Lavaee F. Motaghi D. Jassbi A. R. Jafarian H. Ghasemi F. Badiee P. (2018). Antifungal effect of bark and root extracts of *Punica granatum* on oral *Candida* isolates. *Curr. Med. Mycol.* 4 20–24. 30815613 10.18502/cmm.4.4.382 PMC6386507

[B22] Mansourian A. Boojarpour N. Ashnagar S. Momen Beitollahi J. Shamshiri A. R. (2014). The comparative study of antifungal activity of *Syzygium aromaticum*, *Punica granatum* and nystatin on *Candida albicans*; an in vitro study. *J. Mycol. Med.* 24 e163–e168. 10.1016/j.mycmed.2014.07.001 25442923

[B23] Marques L. Pinheiro A. Araujo J. de Oliveira R. Silva S. Abreu I. (2016). Antiinflammatory effects of a pomegranate leaf extract in LPS-induced peritonitis. *Planta Med.* 82 1463–1467. 10.1055/s-0042-108856 27352385

[B24] Martinez L. R. Casadevall A. (2007). Cryptococcus neoformans biofilm formation depends on surface support and carbon source and reduces fungal cell susceptibility to heat, cold, and UV light. *Appl. Environ. Microbiol.* 73 4592–4601. 10.1128/AEM.02506-06 17513597 PMC1932807

[B25] May R. Stone N. Wiesner D. Bicanic T. Nielsen K. (2016). *Cryptococcus*: from environmental saprophyte to global pathogen. *Nat. Ver. Microbiol.* 14 106–117. 10.1038/nrmicro.2015.6 26685750 PMC5019959

[B26] Maziarz E. K. Perfect J. R. (2016). Cryptococcosis. *Infect. Dis. Clin. North Am.* 30 179–206. 10.1016/j.dc.2015.10.006 26897067 PMC5808417

[B27] Mukherjee P. K. Sheehan D. J. Hitchcock C. A. Ghannoum M. A. (2005). Combination treatment of invasive fungal infections. *Clin. Microbiol. Rev.* 18 163–194. 10.1128/CMR.18.1.163-194.2005 15653825 PMC544182

[B28] Odds F. C. (2003). Synergy, antagonism, and what the chequerboard puts between them. *J. Antimicrob. Chemother.* 2:1. 10.1093/jac/dkg301 12805255

[B29] Paul S. Mohanram K. Kannan I. (2018). Antifungal activity, gas chromatographic-mass spectrometric analysis and in silico study of *Punica Granatum* peel extracts against fluconazole resistant strains of *Candida* species. *Curr. Pharm. Biotechnol.* 19 250–257. 10.2174/1389201019666180515104800 29766800

[B30] Pessoa C. C. B. Silva S. H. M. Gomes F. S. (2012). Produção de fatores de virulência in vitro por isolados de *Cryptococcus neoformans* e *Cryptococcus gattii* de origem clínica em Belém, Estado do Pará, Brasil. *Rev. Pan Amazôn. Saúde* 3 59–65. 10.5123/s2176-62232012000200008

[B31] Pinheiro A. J. M. C. R. Gonçalves J. S. Dourado A. W. A. Sousa E. M. Brito N. M. Silva L. K. (2018). *Punica granatum* L. leaf extract attenuates lung inflammation in mice with acute lung injury. *J. Immunol. Res.* 2018:6879183. 10.1155/2018/6879183 29675437 PMC5838491

[B32] Pinheiro A. J. M. C. R. Mendes A. R. S. Neves M. D. F. J. Prado C. M. Bittencourt-Mernak M. I. Santana F. P. R. (2019). Galloyl-Hexahydroxydiphenoyl (HHDP)-glucose isolated from *Punica granatum* L. leaves protects against Lipopolysaccharide (LPS)-induced acute lung injury in BALB/c Mice. *Front. Immunol.* 10:1978. 10.3389/fimmu.2019.01978 31481965 PMC6710369

[B33] Pinto C. M. F. De Oliveira Pinto C. L. Donzeles S. M. L. (2013). Pepper *Capsicum*: chemical, nutritional properties, pharmacological and medicinal products and their potential for agribusiness. *Rev. Bras. Agrop. Sustent.* 3 108–120.

[B34] Ravi S. Pierce C. Witt C. Wormley F. L. Jr. (2009). Biofilm formation by *Cryptococcus neoformans* under distinct environmental conditions. *Mycopathologia* 167 307–314. 10.1007/s11046-008-9180-6 19130292 PMC4278410

[B35] Rêgo M. F. Rodrigues R. E. F. Do Nascimento W. S. Silva H. M. (2019). Bibliographical analysis of main aspects of cryptococosis. *Braz. J. Health Ver.* 2 3797–3807. 10.34119/bjhrv2n4-141

[B36] Santos J. R. A. César I. C. Costa M. C. Ribeiro N. Q. Holanda R. A. Ramos L. H. (2016). Pharmacokinetics/Pharmacodynamic correlations of fluconazole in murine model of cryptococcosis. *Eur. J. Pharm. Sci.* 92 235–243. 10.1016/j.ejps.2016.05.022 27235581

[B37] Santos J. R. A. Gouveia L. F. Taylor E. L. S. Resende-Stoianoff M. A. Pianetti G. A. César I. C. (2012). Dynamic interaction between fluconazole and amphotericin B AGAINST *Cryptococcus gattii*. *Antimicrob. Agents Chemother.* 56 2553–2558. 10.1128/AAC.06098-11 22290956 PMC3346628

[B38] Silva B. T. Dos Anjos C. Novo S. M. F. Matsumoto L. S. Peixoto E. C. Silva L. P. (2013). In vitro antimicrobial activity of *Punica granatum* L. extract on *Staphylococcus aureus* isolated in bovine milk. *Biosci. J.* 29 974–984.

[B39] Tavares E. R. Gionco B. Morguette A. E. B. Andriani G. M. Morey A. T. Do Carmo A. O. (2019). Phenotypic characteristics and transcriptome profile of *Cryptococcus gattii* biofilm. *Sci. Rep.* 9:6438. 10.1038/s41598-019-42896-2 31015652 PMC6478838

[B40] Worasilchai N. Tangwattanachuleeporn M. Meesilpavikkai K. Folba C. Kangogo M. Groß U. (2017). Chindamporn, diversity and antifungal drug susceptibility of *Cryptococcus* isolates in Thailand. *Med. Mycol.* 55 680–685. 10.1093/mmy/myw130 27915307

